# Hybrid video-assisted thoracoscopic surgery lobectomy of fissureless congenital cystic adenomatoid malformation: a case report

**DOI:** 10.1186/1752-1947-9-23

**Published:** 2015-02-05

**Authors:** Mitsuyuki Nakata, Shigetoshi Yoshida, Takeshi Saito, Keita Terui, Tetsuya Mitsunaga, Sachie Ohno, Naoko Mise, Satoru Oita, Hideo Yoshida

**Affiliations:** 14 Department of Pediatric Surgery, Graduate School of Medicine, Chiba University, 1-8-1 Inohana, Chuo-ku, Chiba, 260-8677 Japan; 15 Department of General Thoracic Surgery, Graduate School of Medicine, Chiba University, 1-8-1 Inohana, Chuo-ku, Chiba, 260-8677 Japan

**Keywords:** Congenital cystic adenomatoid malformation of lung, Hybrid VATS, Thoracic surgery

## Abstract

**Introduction:**

Thoracoscopic lobectomy for congenital pulmonary airway malformation has been indicated from the neonatal period to adolescence. However, it is difficult to approach the pulmonary artery for lobectomy in congenital lung malformations with incomplete or absent interlobar fissures. Multidetector computed tomographic images and computed tomography pulmonary angiography gave us helpful information before the operation. We performed thoracoscopic lobectomy for congenital pulmonary airway malformations with absent interlobar fissures and adhesions in accordance with information from multidetector computed tomographic images.

**Case presentation:**

A 14-year-old Japanese girl received a diagnosis of congenital pulmonary airway malformation when she presented with pneumonia. Using multidetector computed tomography and three-dimensional reconstruction provides meticulous characterization of the anatomy in pediatric patients. We confirmed that her left A4+5 artery arose from her left pulmonary artery medial to A6. Her left pulmonary artery was divided just proximal to the A6 origin before the lobes were separated safely. We took advantage of using a stapler to divide the fissureless thick parenchyma. Perioperative diagnosis was congenital cystic adenomatoid malformation.

**Conclusions:**

We used preoperative multidetector computed tomography to outline the bronchovascular anatomy and guide hybrid video-assisted thoracoscopic surgery for a congenital cystic adenomatoid malformation in a fissureless left lung.

## Introduction

Congenital pulmonary airway malformation (CPAM) has been historically diagnosed in a broad range of age groups, from the prenatal period to adulthood. The spectrum of CPAM requiring surgery includes congenital cystic adenomatoid malformation (CCAM), intra- and extrapulmonary sequestration, and symptomatic congenital lobar emphysema. The indications for surgery of asymptomatic CPAM are controversial [1]. The risk of infection in the first 3 months after birth is low. Stanton *et al*. reported that asymptomatic antenatally diagnosed infants developed symptoms at a median age of 6.9 months (range, 2 to 10 months) [2]. Pelizzo *et al*. reported chronic inflammation in 50% of asymptomatic CPAMs resected at 3 months of age [3]. This suggests that lobectomy in neonates or infants is desirable, even in asymptomatic cases. Although there are still few reports of thoracoscopic lobectomy in children, thoracoscopic lobectomy for CPAM has been indicated from the neonatal period to adolescence [4–6]. However, adhesions and incomplete or absent interlobar fissures make thoracoscopic surgery difficult, even in adults [7].

In this paper, we describe using multidetector computed tomography (MDCT) three-dimensional reconstructions to guide a hybrid video-assisted thoracoscopic surgery (VATS) lobectomy safely in a patient with absence of the interlobar fissure, and provide a brief review of the existing literature.

## Case presentation

**Figure 1:**
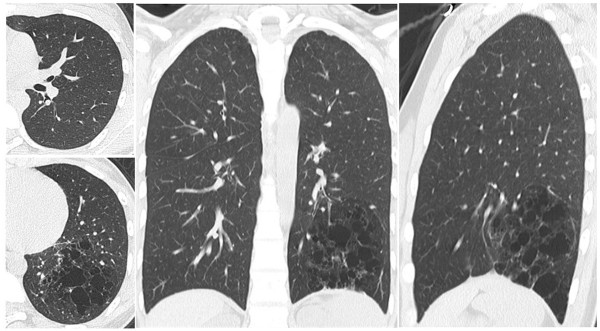
**High-resolution computed tomography of the lung.** Congenital pulmonary airway malformation was detected in the basal segment of left inferior lobe with a severely atretic interlobar fissure. The lesion consisted of multiple cysts with no abnormal vessels.

A 14-year-old Japanese girl received a diagnosis of CPAM when she presented with pneumonia. Volumetric thin-section high-resolution computed tomography (CT) revealed almost complete absence of the left interlobar fissure (Figure 1). A lesion was detected in the basal segment of her left lower lobe, consisting of multiple cysts but no abnormal vessels. The preoperative diagnosis was CCAM.

**Figure 2:**
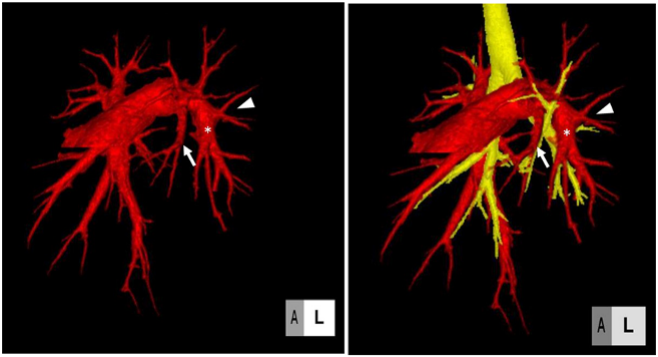
**Three-dimensional computed tomography of the pulmonary artery, trachea and bronchus.** Computed tomographic pulmonary angiography with three-dimensional reconstructions showing the left A4+5 artery (the mediastinal lingual artery) arising medial to A6. Red image, pulmonary artery; yellow image, trachea and bronchus; white arrow, A4+5 (the mediastinal lingual artery); white arrowhead, A6; asterisk, common basal artery.

CT pulmonary angiography with three-dimensional reconstructions of her pulmonary vessels was performed. Images were acquired using a 16-slice scanner (LightSpeed Ultra; GE Healthcare, Milwaukee WI, USA). Iohexol (400mg I/kg of contrast medium, to a maximum of 100mL) was injected intravenously at a rate of 2mL/second. Images revealed her left lingual artery to be the first branch of her left main pulmonary artery, which is the so-called mediastinal lingual artery (Figure 2).

**Figure 3:**
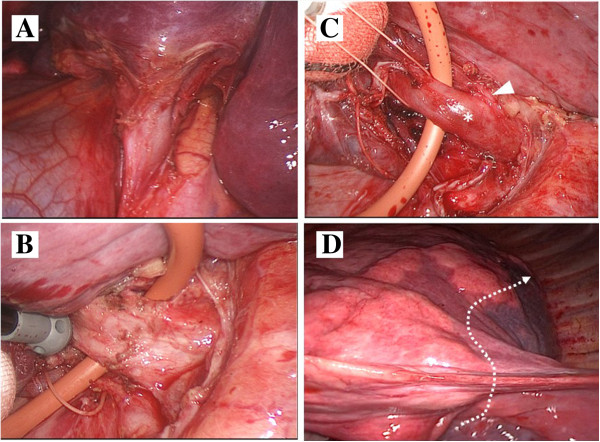
**Operative procedure.** Almost no fissure in the left lung was identifiable after the arteries, veins, and bronchi were divided. After isolating and dividing the left inferior pulmonary vein **(A)**, the lower bronchus **(B)** and the pulmonary artery (**C**: arrowhead, A6; asterisk, common basal artery), the residual lung was inflated to visualize the border with the lower lobe (**D**: white dotted line).

Hybrid VATS lobectomy using differential lung ventilation was performed in the right lateral decubitus position. We used a small thoracotomy incision (5.5cm) along the fifth intercostal space centered on the midaxillary line, and two ports in the sixth intercostal space along the postaxillary line and in the seventh intercostal space along the midaxillary line. As seen on CT, her left lung had almost no fissure. Her left inferior pulmonary vein was first divided using a stapler (Endo-GIA™ white, Covidien, Mansfield MA, USA). It was difficult to free the affected arteries and bronchi from extensive adhesions. After we located the left upper and lower bronchi using a bronchoscope, we confirmed the CT finding of the mediastinal lingual artery arising medial to A6. Before the fissures were separated, the left lower bronchus was divided using a stapler (Endo-GIA purple) and her left pulmonary artery was divided, also with a stapler (Endo-GIA white), just proximal to the origin of A6. After her left lung was inflated by an anesthesiologist, the border line between the deflated and the inflated area clearly appeared (Figure 3). Her left lower lung was divided with staplers (Endo-GIA purple one time and black four times). The procedure lasted 5 hours 22 minutes with a small amount of blood loss. There were no complications.

## Discussion

Thoracoscopic lobectomy for CPAMs in pediatric patients is practical and safe. Patients are generally pleased with the cosmetic result. The good view of the hilar area ensures less blood loss. Nasr and Bass reported that there was no significant difference between thoracoscopic and open procedures in the rate of overall complications and the duration of surgery by meta-analysis [8]. Our operation was hampered by adhesions (probably due to previous infections) [8] and an atretic interlobar fissure.

In the case of incomplete interlobar fissures or fused fissures it is difficult to determine the arterial anatomy, and forcible division of fused fissures risks vessel injury and prolonged air leakage [9].

We selected hybrid VATS. A minithoracotomy combined with video assist performed predominantly via direct visualization was a secure, integrated, minimally invasive approach to the large fused fissure and the severe adhesion [10].

Preoperative imaging studies are essential for assessing not only the congenital lung disease but also the anatomy of vessels, bronchi, and fissures. Using MDCT and three-dimensional reconstruction provides meticulous characterization of the anatomy in pediatric patients. According to Lee *et al*., types and location of congenital lung disease and anomalous vessels associated with congenital lung anomalies were detected with high accuracy [11]. In our case, we knew that the left A4+5 artery was the mediastinal lingual artery and the left pulmonary artery was divided just proximal to the A6 origin before the fissures were separated.

During the operation it may be necessary to produce a fissural separation. Inadvertent separation results in prolonged air leakage. It has been reported that the use of various devices, such as staplers, bipolar thermofusion, biodegradable sealant, and ultrasonically activated scalpels [12] can prevent prolonged air leakage. Automatic stapling devices have been often used for interlobar fissure division for pulmonary lobectomy. Thomas *et al*. explained how to use staplers safely to divide fissures [13]. Using staplers is helpful in dividing thick parenchyma with no fissure. Nomori *et al*. reported that there was no postoperative air leakage in the patients after lobectomy with large fused fissures divided by staplers. They defined the steps of the procedures according to each lobectomy [7]. Thomas *et al*. introduced the technique of dividing the parenchyma with staplers to expose the pulmonary arteries [13].

In neonates and infants the operative field is too small to use a stapler. Albanese *et al*. recommended bipolar electrocoagulation (LigaSure™ Covidien, Mansfield MA, USA) for sealing pulmonary vessels and dividing fissures during lobectomy in infants. In their series, they used this on both arteries and veins less than 7mm in diameter because of the low pressure pulmonary circulation in small children [14]. Santini *et al*. assessed the efficacy and safety of bipolar electrocoagulation in experimental studies with animals and humans; they found it was useful in dividing fissures [15]. Kaneko *et al*. suggested that bipolar electrocoagulation seemed to have a weaker sealing effect under wet conditions, and there was an increased risk of hemorrhage [16].

## Conclusions

In summary, we reported hybrid video-assisted thoracoscopic surgical lobectomy of fissureless CCAM for a 14-year-old girl. Thoracoscopic lobectomy for CPAM has been indicated from the neonatal period to adolescence. However, it is difficult to approach the pulmonary artery for lobectomy in congenital lung malformations with incomplete or absent interlobar fissures. MDCT and CT pulmonary angiography gave us helpful information before the operation. We confirmed that her mediastinal lingual artery arose medial to A6. Her left pulmonary artery was divided just proximal to the A6 origin before the lobes were separated safely. We took advantage of using a stapler to divide the fissureless thick parenchyma. We performed safely hybrid VATS lobectomy on an essentially fissureless lung using staplers, guided by preoperative MDCT and CT pulmonary angiography.

## Consent

Written informed consent was obtained from the patient’s parent for publication of this case report and accompanying images. A copy of the written consent is available for review by the Editor-in-Chief of this journal.
